# Is there such a thing as a 'lope' dope? Analysis of loperamide-related European Medicines Agency (EMA) pharmacovigilance database reports

**DOI:** 10.1371/journal.pone.0204443

**Published:** 2018-10-04

**Authors:** Fabrizio Schifano, Stefania Chiappini

**Affiliations:** 1 Psychopharmacology, Drug Misuse, and Novel Psychoactive Substances Research Unit, School of Life and Medical Sciences, University of Hertfordshire, Hatfield, Herts, United Kingdom; 2 Casa di Cura Villa Rosa, Viterbo (VT), Italy; Chuo University, JAPAN

## Abstract

**Background:**

Among over-the-counter (OTC) drugs, loperamide has recently emerged for its potential of misuse and cardiotoxicity issues. Hence, we aimed here at assessing the loperamide-related cases being reported to the EMA's EudraVigilance (EV) database.

**Methods:**

All spontaneous EV reports relating to loperamide misuse/abuse/dependence/withdrawal and cardiotoxicity issues were here retrieved, performing a descriptive analysis.

**Findings:**

During the years 2005–2017, EV collected a number of 1,983 (out of a total of 7,895; 25.11%) loperamide-related misuse/abuse/dependence/withdrawal adverse drug reaction (ADR) reports, with a progressively increasing trend since 2014. Most cases were classified as drug use disorder (37.4%) or intentional overdose (25.4%). Loperamide was used on its own in 41.9% of cases; remaining, polydrug, cases included antidepressants; benzodiazepines; and other OTCs. Some 1,085 (1,085/7,895 = 13.7%) cardiovascular ADRs were reported, being conduction abnormalities and EKG alterations the most frequently identified.

**Conclusions:**

EV data may support the levels of concern relating to loperamide potential of abuse and associated cardiotoxicity issues.

## Introduction

In being perceived to be safe and appropriate for use without the supervision of a healthcare professional, over-the-counter (OTC) medicines are meant to be delivered without the need for a prescription. As more OTCs become available, concerns regarding their misuse have been increasing [1], with OTC abuse (‘pharming’; [2]) being an internationally recognized issue [3–6]. Even though at the EU-wide level a strong legal framework for licensing, manufacturing and distribution of medicines is available [7], it is typically problematic to quantify actual OTC misuse and abuse levels [8]. Findings of a 2014 Scottish survey reported however that the proportion of pharmacists considering a suspected OTC misuse increased to 80.8% from 70.8% in 2006; codeine-containing products were most frequently perceived to be misused, followed by diphenidramine and laxatives [9]. Conversely, Levine [2] and Finkelstein *et al* [10] suggested increased levels of non-medical use of OTC cough suppressants containing dextromethorphan, whilst stimulant and sleep aid molecules’ misuse has been suggested elsewhere [11–12].

Within the OTC group, the focus was here on loperamide, as levels of concern were recently raised due to the molecule’s euphoric effects; diversion; and use to alleviate opiate/opioid withdrawal [13–15]. Detailed, pro drug, comments on how to best take advantage of the recreational loperamide potential are easily accessible online (e.g., Bluelight [16]; Drugs-forum [17]). Misusers experience their ‘lope highs’ after consuming huge quantities of the molecule, which is at times being described as ‘better than oxycodone’ [18]. Valued as the ‘poors’ methadone’ [19], loperamide has been anecdotally advised online to reduce opiate/opioid withdrawal symptoms [20–21]. Moreover, a growing trend in the number of published cases of loperamide toxicity from 1985 to 2016 has been identified, with more than 50% of such reports published between 2014 and 2016 [22]. Consistent with this, Eggleston *et al* [23] analyzed the January 2008–March 2016 loperamide abuse cases reported to the National Poison Database System (NPDS) and the New York City Poison Control Center (NYCPCC). The number of calls to poison centres for intentional loperamide exposures more than doubled between 2010 and 2015, and approximately 50% of abuse cases were reported after January 2014. In the context of elevated loperamide concentrations, fatalities have been reported as well [24]. The US North Carolina Office of the Chief Medical Examiner recently identified 21 loperamide misuse/abuse cases where the drug was present in supra therapeutic dosages (>16 mg/die). In 19 cases the drug was considered as directly implicated in death and, in most cases, loperamide was self-administered on its own [25]. Vakkalanka *et al* [26] assessed the 2010–2015 National Poison Data System loperamide exposures and identified a 91% increase of cases overtime; loperamide was the only agent in half cases. Lasoff *et al* [27] carried out a 2002–2015 retrospective review of a California (US) poison control system's experience with loperamide misuse/abuse and identified a total of 224 exposures. Although most cases presented as being of minor/moderate severity, 3 fatalities and 9 cardiotoxicity cases were identified. In reviewing the 2009–2015 Texas Poison Center Network's database loperamide misuse/abuse exposure calls, Borron *et al* [28] found that the number of loperamide alone calls nearly doubled, confirming its significant cardiotoxic effects.

Loperamide is a common OTC anti-diarrhoeal compound, considered safe in the 2–16 mg daily dosage range. Within these levels, due to a rapid metabolism and a poor blood–brain barrier penetration, loperamide may be lacking any abuse potential [29]. Loperamide is a potent mu-opioid receptor agonist with predominantly peripheral activity on the myenteric plexus, primarily decreasing intestinal propulsive activity; it reduces daily faecal volume whilst decreasing the loss of fluid and electrolytes. Secondary peripheral effects are seen at κ-opioid and δ-opioid receptors [30]. These receptor activities initially prompted, in 1977, the US FDA to place loperamide in Schedule V of the Controlled Substance Act. Eventual studies [31], however, supported its safety and low physical dependence risk, and by 1988 loperamide was made available for OTC use in the USA. Ingestion of higher, e.g. beyond 50 mg, loperamide dosages has however been associated with euphoria, central nervous system depression, and cardiotoxicity [32–35], recently prompting the FDA to release a safety warning commenting on the safety risks of ingesting high dosages of loperamide [30; 36].

To assess loperamide misuse-; abuse-; dependence-; withdrawal-; and cardiotoxicity-related issues, we aimed here at analyzing the European Medicines Agency (EMA) EudraVigilance (EV) database, which collects electronic reports of suspected adverse drug reactions (ADRs) for all medicinal products authorized in the European Economic Area/EEA; more specifically, focus was here on the August 2005-August 2017 loperamide-related ADRs.

## Methods

The EMA, responsible for the safety monitoring of medicines developed for use in the European Union, is operating through the EV, which manages and analyses information on suspected ADRs to medicines which have been authorized in the European Economic Area (EEA). The EV data analysis system (EVDAS) database here assessed focused on the August 2005-August 2017 case reports relating to both misuse- and cardiotoxicity-related loperamide ADRs. Upon a specific request, the EMA sent a database including all suspected ADRs relating to loperamide as medicinal product (including both generic names and brand names), as reported to the EV dataset. The ADRs here considered were, per se, spontaneous and unsolicited communications [37] reported by both Regulatory Authorities of the EU Member States where the reaction occurred, and/or by the Marketing Authorization Holders for those ADRs occurring outside the EEA. Within the standardized MedDRA Query (SMQ; Version 20.1; 2017) ‘drug abuse, dependence and withdrawal’ section Medical Dictionary for ADRs [38] we identified the following adverse reactions: dependence, drug abuser, drug use disorder, drug withdrawal convulsions, drug withdrawal syndrome, intentional overdose, intentional product misuse, intentional product use issue, overdose, product use in unapproved indication, product use issue, substance use disorder, withdrawal. ‘Misuse’ was here meant to be the ‘intentional use for a therapeutic purpose by a patient or consumer of a product, over-the-counter or prescription, other than as prescribed or not in accordance with the authorized product information’. Conversely, ‘abuse’ was here defined as the ‘intentional, non-therapeutic use by a patient or consumer of a product, over-the-counter or prescription, for a perceived reward or desired non-therapeutic effect including, but not limited to, getting high (euphoria)’. The term ‘addiction’, typically replaced by ‘dependence’, is the ‘overwhelming desire by a patient or consumer to take a drug for non-therapeutic purposes together with inability to control or stop its use despite harmful consequences’. Finally, ‘withdrawal’ referred here to: ‘a substance-specific syndrome which follows cessation or reduction in the intake of a psychoactive substance previously regularly used’ [39]. For each ADR, the online [40] information made available relating to the suspected side-effect reports was limited to the following issues: ‘seriousness’; ‘geographic origin’, ‘reporter group’, ‘sex’, ‘age group’, ‘reaction groups”, and ‘reporter suspected reaction’. In order to improve the knowledge relating to each case, consistent with current ongoing policies and regulations EMA provided us with access to EV data through a dedicated hyperlink. All Individual Case Safety Reports (ICSR) were retrieved from the post-authorization modules (EVPM) backlog; the ICSRs were selected through Preferred Terms (PTs), which included the following: ‘drug abuse’, ‘intentional product misuse’, ‘drug dependence’ and ‘withdrawal syndrome’ and all remaining PTs which are part of the broader ‘drug abuse, dependence and withdrawal’ SMQ. Furthermore, to assess the possible occurrence of cardiotoxicity issues, the request focused on the following PTs: ‘cardiotoxicity’; ‘conduction abnormalities’; ‘EKG alterations’; ‘syncope’; ‘hypotension’; ‘cardiac arrest’; ‘cardiorespiratory arrest’.

Level 2A EV frequency table and line listing of the requested ADRs were here retrieved. In the EV database each individual patient had a code (EV local number) for the unequivocal identification. ADRs’ numbers differed from those referring to single patients, since different reporters/senders could have independently flagged the same ADR to EMA, or several ADRs (involving various organ classes and hence identified with specific PTs) related to the primary searched ADRs (abuse/misuse/dependence and withdrawal ADRs) for the same patient could have been reported as well. Patients were analyzed considering a range of parameters, including socio-demographic characteristics (age and sex); source/reporter geographical origin; country and reporter qualification (i.e. pharmacist, physician); outcomes (fatal, recovered, resolved); loperamide dosage; and possible concomitant drug(s). Suicides were here reported as ‘suicide attempt’ and as ‘intentional self-injury’. ‘Overdose’, including “intentional overdose”, was not interpreted here as being a suicidal attempt [38].

### Ethics’ issues

Because of EMA protection of privacy and integrity of individuals, certain data elements (e.g. names/identifiers of individuals involved; country-specific information, nationally authorized products etc) were here not disclosed. The study was approved by the University of Hertfordshire Ethics' Committee (reference number LMS/PGR/UH/03234).

## Results

A total (e.g. all categories) of 10,991 loperamide–related ADRs were reported to EV between August 2005 and August 2017; out of these, 7,895 (71%) were classified as ‘suspect’ and were here properly analysed (Table 1).

**Table 1:** Overview of loperamide misuse-abuse-/dependence-/withdrawal-related ADRs as reported to the EV database.

	LOPERAMIDE ADRs
**Time-frame considered**	08/2005–08/2017
**Total number of ‘suspect’ ADRs**	7,895
**Misuse-abuse-/dependence-/withdrawal-related ADRs**	**1,983 (1,983/7,895 = 25.11%)**
**Number of unique patients reported to the database**	434
**Age-range most typically represented**	18–64 yy (4,577/ 7,895 = 57.9%)
**ADRs most typically represented**	Drug use disorder 742 (742/1,983 = 37.4%) Intentional overdose 502 (502/1,983 = 25.3%) Intentional product misuse 296 (296/1,983 = 14.9%)
**Gender most typically represented**	Female (F/M ratio:4,401/3,397 = 1.29)
**Loperamide identified as the sole drug**	182 cases (182/434 = 41.9%)
**Concomitant drugs most typically represented in the remaining (434–182) 252 cases**	Antidepressants in 44 cases (44/252 = 17.5%); SSRIs most typically reported; Benzodiazepines in 40 cases (40/252 = 15.9%); Opioids in 23 cases (23/252 = 9.13%); Other psychotropic drugs in 21 cases (21/252 = 8.3%); Antipsychotics in 11 cases (11/252 = 4.36%); Mood stabilizers in 9 cases (9/252 = 3.57%)
**Resulted in death**	305/1,983 (15.34%, corresponding to 94/434 cases: 21.6%)
**Suicides**	373 ADRs, corresponding to 42/434 cases; 9.67%

The number of ADRs remained flat until 2014, and then peaked in 2015 (853 ADRs), 2016 (931 ADRs) and 2017 (3,867 ADRs until August 2017). Most ADRs involved adult females (female/male ratio: 1.29) and were reported by pharmaceutical companies (67.8%) which were typically located in non-EEA countries (64.1%), and especially in North America. Out of the total number of 7,895 suspect ADRs, the misuse/abuse/dependence/withdrawal ADRs resulted to be 1,983 (25.11%; relating to 434 unique subjects), with ‘drug use disorder’ (37.4%), ‘intentional overdose’ (25.3%), and ‘intentional product misuse’ (14.9%) being the most represented ADRs (Table 2).

**Table 2:** Overview of all 2005–17 loperamide misuse/abuse-; dependence-; and withdrawal–related ADRs reported to the EMA.

LOPERAMIDE ADRs		No of reactions ADRs
**Misuse-; abuse; and overdose-related ADRs (n = 1,861)**	**Drug use disorder**	**742 (742/1,983 = 37.4%)**
	**Intentional overdose**	**502 (502/1,983 = 25.3%)**
	**Intentional product misuse**	**296 (296/1,983 = 14.9%)**
	Overdose	152 (152/1,983 = 7.6%)
	Intentional product use issue	102 (102/1,983 = 5.1%)
	Product use in an unapproved indication	40 (40/1,983 = 2%)
	Product use issues	8 (8/1,983 = 0.4%)
	Substance use disorder	14 (14/1,983 = 0.70%)
	Drug abuse	5 (5/1,983 = 0.25%)
**Dependence-related ADRs (n = 8)**	Dependence	8 (8/1,983 = 0.4%)
**Withdrawal-related ADRs (n = 112)**	Withdrawal	27 (27/1,983 = 1.36%)
	Drug withdrawal syndrome	79 (79/1,983 = 4%)
	Drug withdrawal convulsions	8 (8/1,983 = 0.4%)
**Total ADRs**		**1,983 (100%)**

Loperamide was reported as having been ingested, in the 2-800mg range, as the sole drug in 182/434 (41.9%) cases. When data were made available, loperamide dosages have been reported beyond 16 mg in 48 cases. Most frequently mentioned compounds in polydrug cases included antidepressants, benzodiazepines, and opioids. However, a range of medications known to increase loperamide effects was here reported as well and included: dextromethorphan (25 cases), diphenidramine (20 cases), cimetidine (13 cases), quinidine-quinine (5 cases), and omeprazole (3 cases). Cardiovascular ADRs (Table 3) were here identified in 1,085/7,895 cases (13.74%), and included: conduction abnormalities/EKG alterations (e.g. tachycardia, ventricular tachycardia, ‘torsades de pointes’; and increased QTc levels); loss of consciousness/syncope; diastolic hypotension; hypokalaemia; and cardiac arrest.

**Table 3:** Overview of data relating to loperamide cardiovascular ADRs.

LOPERAMIDE Suspect CV ADRs: 1,085/7,895 (160/434 unique cases)	No of reactions/ADRs
**CONDUCTION ABNORMALITIES** (Tachycardia, Ventricular Tachycardia, Torsades de Pointes, Arrhytmias, Ventricular Arrythmias/Fibrillation, Conduction disorder, (Sinus) Bradycardia, AV block (I-II degree), Brugada Syndrome, HR irregular/increased/ decreased, Defect Conduction Intraventricular)	**494** (494/1,085 = 45.5%)
**EKG ALTERATIONS** (EKG QT prolonged, QRS prolonged/abnormal/ shortened, long QT syndrome, EKG PR prolongation/shortened, EKG P abnormal, EKG ST-T change)	**322** (322/1,085 = 29.7%)
**LOSS OF CONSCIOUSNESS, SYNCOPE, HYPOTENSION**	**97** (97/1,085 = 8.9%)
**CARDIAC ARREST/CARDIORESPIRATORY ARREST/ SINUS ARREST**	**77** (77/1,085 = 7.1%)
**HYPOKALAEMIA**	**76** (76/1,085 = 7%)
**CARDIOTOXICITY**	**11** (11/1,085 = 1%).

### Fatalities

Out of the 1,983 misuse/abuse/dependence/withdrawal ADRs, fatalities were reported in 305 ADRs (15.34%, corresponding to 94/434 cases: 21.6%). Most fatalities involved adult males and were related to the following ADR categories: drug use disorder (65.7%), intentional product misuse (17.1%), overdose (8.57%), and accidental overdose (28.57%). In roughly 1 out of 2 fatalities, a polydrug misuse ingestion was identified, with the molecules most typically mentioned including: benzodiazepines (20 cases); antidepressants (17 cases); dextromethorphan (12 cases); opioids (9 cases); mood stabilizers (6 cases); illicit recreational drugs (e.g., amphetamines, cannabis, nicotine, alcohol, caffeine; 5 cases); and antipsychotics (3 cases).

A suicidal episode was specifically mentioned in 373 ADRs, corresponding to 42/434 (9.7% unique cases). Multi-drug toxicity was reported in 39/42 suicidal cases. When reported, most cases involved the oral ingestion of loperamide dosages in excess of 30, and up to 800, mg.

Finally, within the sub-group of 1,085 cardiovascular ADRs (160 cases), 34 ADRs (15 cases; 9.4%) resulted to be fatal. Deaths were related to cardiac/cardiorespiratory arrest, arrhythmias and cardiotoxicity. In 12 out of those 15 cases, loperamide was reported on its own, and in 1 case in combination with dextromethorphan.

## Discussion

This unique, large scale, study aimed at systematically identifying and analysing loperamide misuse/abuse/dependence/withdrawal and cardiotoxicity issues. Present data were extracted from a high-quality and large-scale pharmacovigilance database, such as the EMA’s EV. Together with the World Health Organization’s Drug Monitoring Programme, the EV database is considered a worldwide reference standard [41]. Most literature papers, so far, were based on small case series/single case studies [13–15]. Conversely, current findings referred to much larger (e.g. 1,983 ADRs; 434 patients) numbers of patients presenting with loperamide misusing issues.

Overall, the misuse/abuse/dependence/withdrawal ADRs were here associated with supra therapeutic, or even extraordinarily high (e.g. up to 20–50 times the 16 mg maximum therapeutic levels) dosages. Although there may be no straightforward explanations for these results, the opiatergic, high dosage intake, loperamide activities [32] may well be associated with its possible recreational value.

After many years of stability, loperamide ADRs seemed to have peaked here since 2015, with this finding being fully consistent with previous reports [26–28]. It is possible that these trends had just mirrored the increasing rates of worldwide availability of this molecule. However, recent, consistently increasing, rates of loperamide misuse/abuse may have been facilitated as well by the high number of pro drug websites offering proper advice on how to best enjoy the ‘lope dope’ experience [27].

To better understand present findings, the United Kingdom (UK) loperamide Drug Analysis Profile (DAP) made available by the Regulating Medicines and Healthcare Products Regulatory Agency (MHRA) through the Yellow Card Scheme [42], was here taken into account as well. A total number of 497 ADR reports and some 1,009 reactions were recorded during the period 1975–2018. Reports doubled from 2014 to 2017. Among the 1,009 total reactions recorded, we found a total of 46 misuse/abuse/dependence/withdrawal loperamide-related reactions, including the following: polydrug interaction and toxicity 17; overdose 8; product use issues 9; substance related and addictive disorders 6; off-label use 3; withdrawal 2; and intentional product misuse 1. Furthermore, the US Food and Drug Administration (FDA) Adverse Event Reporting System (FAERS) database, containing adverse event reports, medication error reports and product quality complaints resulting in adverse events, was here considered as well. According to the FAERS [43], a number of 12,851 loperamide cases were recorded during the 1977–2018 time-frame. Reports showed increasing levels overtime, starting in 2007 and peaking in both 2015 and 2017, when recorded numbers identified were respectively 2,019 and 2,010. Out of these 12,851 cases, drug/substance abuse; drug diversion; intentional product use/misuse issues; and drug/substance use disorder reports were identified in 1,204 (9.37%) cases, with dependence-; withdrawal- and tolerance-related reports having been reported in 89 cases. Out of these 1,204 cases, 671 were considered as being ‘serious’, and included mentions of cardiovascular issues; 252 fatalities were identified as well. One might conclude that an overall consistency, both in terms of types of reactions being reported and peaks in reporting, appeared here between current data and those identified by both the MHRA and the FDA.

Apart from benzodiazepines and opiates/opioids, antidepressants (mostly SSRIs) were those drugs most frequently identified in combination with loperamide. Whilst this may suggest the comorbid presence of depression in these patients, SSRIs have been identified as P-glycoprotein (P-gp) inhibitors [44], hence increasing loperamide bioavailability levels. Loperamide is a substrate for P-gp; this is an ATP-binding efflux transporter acting as a cell membrane extruder [45], hence increasing the elimination of xenobiotics from the central nervous system whilst protecting the body from potentially harmful substances [46]. Oral loperamide ingestion is characterized by less than 2% bioavailability levels [46], and, when loperamide is taken as advised, any potential P-gp inhibition involvement is unlikely to become problematic for the user [45–46]. Conversely, large loperamide dosages or its combination with a molecule that will slow down the effectiveness of P-gp will produce a ‘great high’. Misusers’ perceived different euphoric effects may be related as well to differences in P-gp expression and activity [47–48].

Consistent with previous reports [13; 29; 49–50], a further range of molecules was here identified, including dextromethorphan, diphenidramine, cimetidine, quinidine-quinine, and omeprazole. Once again, it is possible that the identification of these molecules in loperamide cases was the result of comorbid medical conditions. These idiosyncratic combinations may however ‘boost’ loperamide effects and hence increase the likelihood of adverse events, including overdose or death [13; 29–30; 49–50]. The OTC cough and cold medication dextromethorphan (DXM) is an opiate/opioid drug, hence arguably synergistically interacting with loperamide. DXM presents with sedative, dissociative, and stimulant properties which can be, at high dosages, of recreational value [2; 51–53]. The antihistamine/diphenidramine intake may have occurred here for its sedative properties, often useful to cope with possible opiate/opioid withdrawal. Both cimetidine and omeprazole are frequently mentioned in pro drug web fora [48] as being able to impact on P-gp activities and hence facilitating the occurrence of the ‘lope ‘highs’ [25]. Since loperamide metabolism is related to cytochrome P450 (CYP450), CYP2C8 and CYP3A4 isozymes [49], its concomitant use with CYP3A4 (e.g. cimetidine, omeprazole, grapefruit juice, tonic water, itraconazole); and CYP2C8 (e.g. gemfibrozil) inhibitors can increase loperamide plasma levels [49; 54]. Finally, the loperamide/quinine-quinidine combination inhibits P-gp activities, hence increasing loperamide bioavailability levels [45–46]. However, quinidine intake is also associated, per se, with QTc prolongation [49–50; 54], further increasing the cardiotoxicity risk. Interestingly, a dextromethorphan/quinidine compound has recently been approved by the US FDA, with quinidine serving to inhibit the CYP2D6 enzymatic degradation of dextromethorphan and thereby increase its circulating concentrations [55].

Cardiovascular ADRs, typically including conduction abnormalities; increased QTc levels; hypotension; and cardiac arrest; were here identified in 1,085 cases (13.7% of the total). This ADRs’ subgroup corresponded to 160 unique patients, and 15 (9.4%) of them died mostly as a result of cardiac/cardiorespiratory arrest and serious arrhythmias. In 12 out of those 15 cases, loperamide was the sole drug being reported. Both loperamide and one of its metabolites (e.g. desmethyl-loperamide) seem to play a role in the ventricular arrhythmias’ pathogenesis [56]. Current findings fully support previously reported loperamide cardiotoxicity concerns [56–57]. Indeed, supra therapeutic dosages may be associated with cardiac arrest, Brugada syndrome [29], recurrent syncope, ventricular tachycardia, marked QT-interval prolongation that may lead to the ‘torsades de pointes’ phenomena, as well as prolongation of the QRS complex [57–59]. The loperamide high-affinity inhibition of sodium channels and a dysfunction of the human ether-a-go-go-related gene (hERG), encoding the pore-forming subunit of the rapidly activating delayed rectifier potassium channel (IKr), could explain both QRS and QT prolongation [58; 60–61]. Occurrence of loperamide-related QT prolongation may be facilitated as well by a range of factors, including: advanced age; co-ingestion with other drugs (e.g. Class 1A and Class III antiarrhythmics; antipsychotics; antibiotics; methadone) that are known to prolong the QT interval [59; 62]; electrolyte abnormalities; and history of: congenital long QT syndrome [30; 56; 62–67].

A number of fatalities had already been reported in the context of elevated (e.g. 195–1600 mg per day dosages) loperamide intake [24; 25; 27; 63]. However, present figures seem to be a reason of concern, since lethal outcomes were here represented in 94/434 (21.6%) cases of patients reported to have misused/abused with loperamide. In about half of these fatalities, loperamide abuse had occurred in combination with a range of prescribing/non prescribing/recreational psychotropics; conversely, multi-drug toxicity was reported in 39/42 (93%) of suicides.

### Limitations

The number of any medicinal compound-related ADRs depends from wide awareness of a safety concern; its market availability levels; extent of use; and serious/non-serious nature of the reaction. Being loperamide an OTC drug, convincing consumption data, to serve as a denominator, are not immediately available. Suspected ADRs alone are rarely sufficient to conclusively prove that a given reaction has been caused by a specific medicine; this could be a symptom of another illness, or it could be associated with another medical product taken by the patient at the same time. Also, due to the nature of EMA EV spontaneous reports, not all data fields are consistently provided. Based on the current reporting rules in the EEA, report duplications or even triplications are possible, i.e. the same ADR could be reported by different healthcare professionals (physicians, pharmacist, nurses etc). Finally, the EMA has to ensure that the protection of privacy and integrity of individuals is guaranteed. Hence, certain data elements (i.e. country-specific information, nationally authorized products) were here not disclosed. Finally, the EV database did not provide here further details of clinical interest, including the possible concurrence of psychopathological conditions.

## Conclusions

Despite loperamide innocuous nature at therapeutic dosages, current findings seem to emphasize the possible health consequences associated with its high dosage intake. Considering the growing loperamide misusing/abusing levels; the lack of detection in routine drug screens; and the challenges in contrasting any OTC ‘pharming’ practice [2; 26], increased awareness is essential toward prevention, diagnosis, and treatment. Physicians should remain vigilant for loperamide abuse, especially considering its cardiovascular toxicity, which is still not adequately characterized [35; 68]. A range of strategies may minimize OTC associated harms [53]. Wright *et al* [9] described individual pharmacy policies regarding drug sales, such as hiding or storing product out of sight; not stocking products; and alerting customers to the abuse potential of products. Pharmacists should refer customers to their GP, or better to specialist addiction services, considering that long-term, high dosage, loperamide misuse/abuse may be managed with methadone [69].
